# Comparison of Visual and Refractive Outcomes Between Refractive Lens Exchange and Keratorefractive Lenticule Extraction Surgery in Moderate to High Myopia

**DOI:** 10.3390/diagnostics15010043

**Published:** 2024-12-27

**Authors:** Chia-Yi Lee, Shun-Fa Yang, Hung-Chi Chen, Ie-Bin Lian, Jing-Yang Huang, Chao-Kai Chang

**Affiliations:** 1 Institute of Medicine, Chung Shan Medical University, Taichung 40201, Taiwan; 2 Nobel Eye Institute, Taipei 10041, Taiwan; 3 Department of Ophthalmology, Jen-Ai Hospital Dali Branch, Taichung 41265, Taiwan; 4 Department of Medical Research, Chung Shan Medical University Hospital, Taichung 40201, Taiwan; 5 Department of Ophthalmology, Chang Gung Memorial Hospital, Linkou, Taoyuan 33305, Taiwan; 6 Center for Tissue Engineering, Chang Gung Memorial Hospital, Linkou, Taoyuan 33305, Taiwan; 7 Department of Medicine, Chang Gung University College of Medicine, Taoyuan 33305, Taiwan; 8 Institute of Statistical and Information Science, National Changhua University of Education, Chunghua 50007, Taiwan; 9 Department of Optometry, Da-Yeh University, Chunghua 51591, Taiwan

**Keywords:** keratorefractive lenticule extraction, refractive lens exchange, astigmatism, uncorrected distance visual acuity, spherical equivalent

## Abstract

**Background/Objectives**: To evaluate the visual and refractive outcomes of keratorefractive lenticule extraction (KLEx) surgery and refractive lens exchange (RLE) surgery in moderate to high myopia patients. **Methods**: A retrospective cohort study was performed, and patients receiving KLEx or RLE surgeries with myopia within −3.00 to −10.00 diopter (D) were enrolled. A total of 19 and 35 patients were put into the RLE and KLEx groups after exclusion. The main outcomes are postoperative uncorrected visual acuity (UDVA), the spherical equivalent (SE), and residual astigmatism via vector analysis. Fisher’s exact test and the Mann–Whitney U test were utilized for the statistical analysis. **Results**: The percentages of patients who reached UDVA results of more than 20/25 and 20/20 were statistically similar between groups (both *p* > 0.05), and the percentages of patients who reached SE results within ±0.50 D and ±1.00 D were statistically similar between groups (both *p* > 0.05). The change in SE in the KLEx group was lesser compared to that in the RLE group (*p* = 0.021). The vector analysis showed a lower DV and ME and a higher CoI in the KLEx group than in the RLE group (all *p* < 0.05). The percentage of patients who reached specific UDVA and SE thresholds were statistically similar between groups with different myopia degrees (all *p* > 0.05). **Conclusions**: The postoperative visual and refractive outcomes between RLE and KLEx surgeries are grossly comparable, while the KLEx may have a slight advantage in astigmatism correction.

## 1. Introduction

Refractive surgeries have been used to correct myopia and astigmatism for more than 20 years [1,2]. Both photorefractive keratectomy and laser in situ keratomileusis have been performed on many people, and the postoperative outcomes are fair [2]. In detail, a postoperative uncorrected distance visual acuity (UDVA) of 20/20 has been found in more than 70 percent of individuals receiving the above two refractive surgeries [3,4]. Still, postoperative complications like ocular pain and residual refractive error have been found to result from these keratorefractive surgeries [5].

Keratorefractive lenticule extraction (KLEx) is one keratorefractive surgery, which was first performed around 2010 [6,7,8,9,10]. Compared to the previous keratorefractive surgeries, the main benefits of KLEx are the small incision and lower risk of postoperative dry eye disease [11,12]. Regarding the visual and refractive outcomes, the first-generation KLEx presents similar UDVA results to both laser in situ keratomileusis and photorefractive keratectomy [13,14,15,16]. The second generation of KLEx surgery has been applied for more than one year and has the advantage of faster laser emission speed compared to the first-generation KLEx surgery [17,18,19]. The postoperative visual outcomes of the second-generation KLEx surgery were similar to those of the first-generation KLEx surgery, while the management of astigmatism was marginally better than with first-generation KLEx surgery [20,21].

In contrast to keratorefractive surgery, refractive lens exchange (RLE) is another method to correct refractive errors [22]. RLE has commonly been applied in those warranting refraction correction, and the usage of RLE in moderate to high refractive error situations can result in fair visual and refractive outcomes [23]. Still, a comparison of the postoperative outcomes between the second-generation KLEx surgery and RLE surgery has not been illustrated. Because the correction methods between the two surgeries are different [18,22], the refractive outcomes may also be different.

Accordingly, the aim of this study is to evaluate the postoperative visual and refractive outcomes of the second-generation KLEx surgery and RLE surgery. Analyses of individuals with different baseline myopia degrees were also performed. The comparison of outcomes between corneal and lenticular refractive surgeries may provide some valuable information for the choice of refractive correction method in the middle-aged population.

## 2. Materials and Methods

### 2.1. Case Selection

This retrospective cohort study was organized at the Nobel Eye Institute, which operates more than 20 clinics in Taiwan. Individuals were selected for this study if they (1) were aged from 20 to 50 years old, (2) had myopia higher than −3.00 diopter (D) but lesser than −10.00 D in cycloplegia refraction, (3) received RLE surgery or the second-generation KLEx surgery at the Nobel Eye Institute, and (4) were followed up on at the Nobel Eye Institute after the surgery for three months or more. Regarding the choice of surgical method, we arranged RLEs in this middle-aged population mostly because they had mild cataracts that did not significantly influence their visual acuity, but these patients' conditions warranted refraction correction. In addition, a minor portion of the patients decided to receive RLE rather than second-generation KLEx surgery because they did not want to receive another eye surgery soon, since cataracts may develop within 5–10 years, considering their age. Furthermore, a few patients had thin corneas, which prevented the arrangement of second-generation KLEx surgery. All the advantages and disadvantages of both surgeries were explained by surgeons to the patients, and refractive surgery was arranged unless patients refused it, and no contraindication was found by surgeons. Also, the following exclusion criteria were determined to exclude the unsuitable cases/eyes: (1) a baseline corrected distance visual acuity (CDVA) lower than 20/40, (2) the presence of any prominent ocular disorders including but not limited to corneal diseases, retinal diseases (retinal degeneration, retinal break, retinal detachment, macular pucker, proliferative diabetic retinopathy, etc.), glaucoma, uveitis, eyelid disorder, optic nerve disorder, and ocular trauma, (3) unstable refraction, which means alteration of more than ±0.50 D in the previous year, (4) pregnancy in the last year, and (5) a monovision (planning residual myopia) approach. Only the right eye of each individual was selected for analysis in this study. After the whole selection process, totals of 19 and 35 eyes were placed into the RLE group and KLEx group, respectively.

### 2.2. Surgery Pattern

All the second-generation KLEx surgeries were performed by one experienced refractive surgeon (C.-K.C.), and all the RLE surgeries were performed by one experienced cataract surgeon (C.-Y.L.). To address the issue of presbyopia in this population, we implanted a presbyopia-correcting intraocular lens in all the patients receiving RLE, and the monovision approach was utilized in those receiving second-generation KLEx surgery (if the right eye was the monovision eye, the patient was excluded, as we stated in the exclusion criteria discussion). The second-generation KLEx surgery was executed by a femtosecond laser device (Visuamax 800, Carl Zeiss, Göschwitzer Str., Jena, Germany). The optic zone of our second-generation KLEx surgery was 5.5-6.9 mm, and the 3.0 mm incision was made at 105 degrees. The angle kappa was revealed by the built-in software of the Visuamax 800, and the data were derived from optical biometry (IOL Master 700, Carl Zeiss, Göschwitzer Str., Jena, Germany). The coaxial sight corneal light reflex and corneal topography were also applied to refine the centration. The cornea was fixated by the suction ring after our centration process; then, the femtosecond laser of the Visumax 800 discharged. Then, a spatula was applied to divide the two interfaces of the corneal lenticule, and the corneal lenticule was then removed by forceps. Concerning the RLE surgery, one phacoemulsification device (Quatera, Carl Zeiss, Göschwitzer Str., Jena, Germany) was utilized. The main incision was made at a superior site, and the ophthalmic viscoelastic device was injected. After performing the continuous curvilinear capsulorhexis, hydrodissection was applied before side-port creation. The phaco-chop technique was performed to clear the nucleus, and the residual cortex was cleaned by an infusion–aspiration probe. One type of extended depth-of-focus intraocular lens (AT LARA^®^, Carl Zeiss, Göschwitzer Str., Jena, Germany) was implanted into the bag, and the rest of the ophthalmic viscoelastic device was sucked out by an infusion–aspiration probe. Finally, the hydroseal technique was performed to seal the main incision and side port; then, tobradex ointment was instilled into the wound area.

### 2.3. Ophthalmic Exam

All the subjects received identical ophthalmic exams in all clinics of the Nobel Eye Institute. The preoperative evaluations included the CDVA, sphere power, and cylinder power via cycloplegia refraction with the aid of an autorefractor (KR-8900, Topcon, Itabashi-ku, Tokyo, Japan). The central corneal thickness (CCT), keratometry (K), corneal astigmatism, and pupil diameter were obtained by a topographic instrument (TMS-5, Tomey Corporation, Nagoya, Aichi, Japan). The pupil diameter and angle kappa were retrieved via a biometry machine (IOL Master 700, Carl Zeiss, Göschwitzer Str., Jena, Germany). The Schirmer I test with topical anesthesia was also completed before the two surgeries. The postoperative evaluations were UDVA, intraocular pressure, and sphere and cylinder powers via the same devices as those used for the preoperative tests. Ophthalmic exams were performed before the surgery, one day after surgery, one week after surgery, one month after surgery, and three months after surgery. The spherical equivalent (SE) refers to the sphere power plus the half-cylinder power.

### 2.4. Statistical Analysis

SPSS version 20.0 (SPSS Inc., Chicago, IL, USA) was utilized for statistical analysis in this study. The Shapiro–Wilk test was utilized to survey the normality of the study populations, and not-normal distributions were found in all data (all *p* < 0.05). Furthermore, the statistical power of this study was 0.69, with an alpha value of 0.05 and a medium effect size, which was calculated by G*power version 3.1.9.2 (Heinrich Heine Universität at Düsseldorf, Germany). A descriptive analysis was performed to reveal the age, sex, cycloplegia refraction, topographic information, and biometric information of the two groups, and then Fisher’s exact test or the Mann–Whitney U test was utilized to survey the differences in the indexes between groups according to their characters. Fisher’s exact test was also utilized to investigate the ratio of postoperative UDVA and SE that reached special checkpoints between the RLE and KLEx groups three months postoperatively. For the trends of UDVA and SE recovery in the postoperative interval, a generalized linear mixed model was utilized to survey the difference between the RLE and KLEx groups after adjusting for age, sex, baseline CDVA, and baseline cycloplegia refraction. Then, the study population was divided into a high myopia subgroup and a low myopia subgroup according to the SE value being higher or lower than −6.00 D, and Fisher’s exact test was utilized again to calculate the differences, regarding whether eyes reached specific points of postoperative UDVA as well as SE, between the two groups. For astigmatism, a vector analysis via the Alpins approach was also utilized to survey target-induced astigmatism (TIA), the magnitude of error (ME), the angle of error (AE), surgically induced astigmatism (SIA), the correction index (CoI), and the difference vector (DV) of the two groups by the Mann–Whitney U test. TIA, SIA, and the DV were revealed as arithmetic means. A *p* value < 0.05 was interpreted as statistically significant; a *p* value over 0.999 was interpreted as *p* > 0.999, and a *p* value under 0.001 was interpreted as *p* < 0.001 in this study.

## 3. Results

The demography of the two groups is presented in Table 1. The mean age was 48.29 ± 8.37 and 42.53 ± 7.52 years in the RLE and KLEx groups (*p* = 0.019). The sex and systemic disease ratios between the two groups also demonstrated insignificant differences (both *p* > 0.05). The preoperative CDVA was 0.05 ± 0.06 in the RLE group, which was worse than that in the KLEx group (*p* = 0.016). The other preoperative parameters, including cycloplegia refraction, demonstrated statistical similarities between the two groups (all *p* > 0.05) (Table 1).

**Figure 1:**
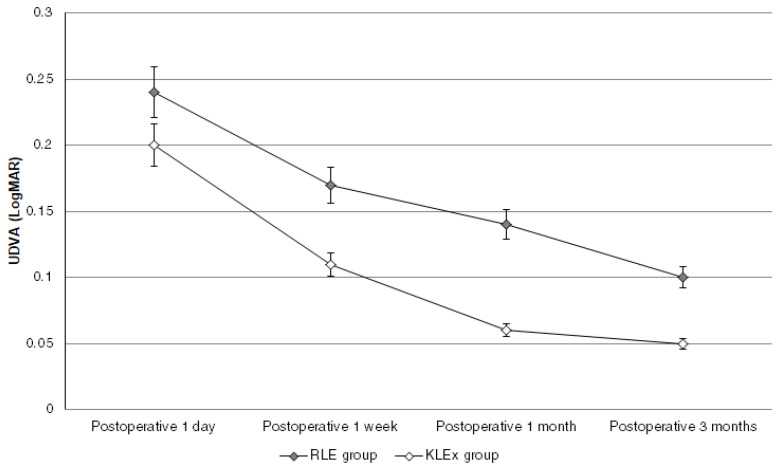
The changes in postoperative uncorrected distance visual acuity of the groups.

**Figure 2:**
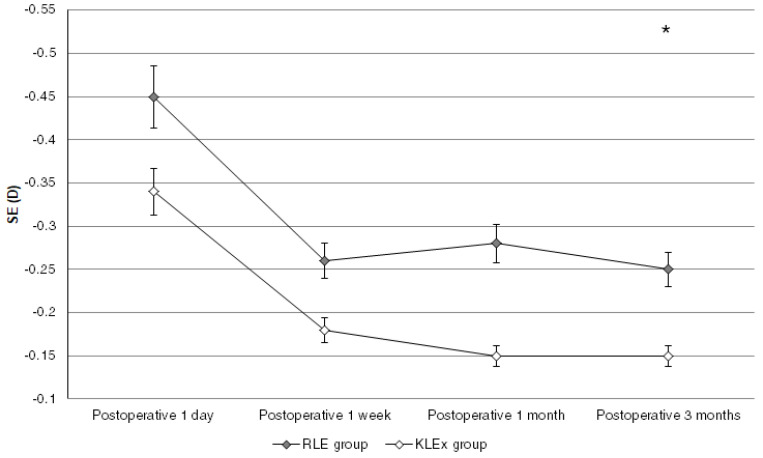
The changes in the postoperative spherical equivalent of the groups. * denotes a significant difference between groups.

The mean final UDVA was 0.10 ± 0.11 and 0.05 ± 0.12 in the RLE and KLEx groups, respectively (*p* = 0.159). Also, the mean final SE was −0.25 ± 0.20 and −0.15 ± 0.17 in the RLE and KLEx groups, respectively (*p* = 0.078). The percentages of patients to reach an UDVA of more than 20/25 and 20/20 were statistically similar between the RLE and KLEx groups (both *p* > 0.05), and the percentages of patients to reach an SE within ±0.50 D and ±1.00 D were statistically similar between the RLE and KLEx groups (both *p* > 0.05) (Table 2). The trends of UDVA changes after the surgery of the two groups were similar (*p* = 0.376) (Figure 1), while the change of SE in the KLEx group was lesser compared to the RLE group (*p* = 0.021) (Figure 2). The vector analysis showed a lower DV, ME, and a higher CoI in the KLEx group than the RLE group (all *p* < 0.05), while the other parameters in the vector analysis were similar between the RLE and KLEx groups (all *p* > 0.05) (Table 3).

In the patients with high myopia, the UDVA one day postoperative was numerically better in the RLE group than the KLEx group (0.25 versus 0.27), while the UDVA one day postoperatively in the low myopia group was numerically better in the KLEx group than the RLE group (0.14 versus 0.23). The percentages of patients to reach UDVA values of more than 20/25 and 20/20 were statistically similar for all the RLE and KLEx patients with different degrees of myopia (all *p* > 0.05), and the percentages of patients to reach SE values within ±0.50 D and ±1.00 D were statistically similar for the RLE and KLEx patients with different degrees of myopia (all *p* > 0.05) (Table 4). There was no newly developed retinal degeneration, retinal break, or retinal detachment episode in any eyes after RLE and second-generation KLEx surgery within a mean follow-up period of 11.5 months (range from 10 to 14 months).

## 4. Discussion

In brief, the postoperative outcomes regarding UDVA and SE of the RLE and KLEx surgeries were similar. Moreover, the astigmatism correction abilities of the two surgeries were similar, although the KLEx surgery represented lower degrees of DV and ME. On the other hand, the final UDVA and SE results were similar for the RLE and KLEx surgeries with different myopia extents.

The postoperative visual acuity comparison between the RLE and KLEx groups illustrated a non-significant difference. In an earlier publication, good postoperative visual acuity was observed in individuals who received KLEx surgery [24]. In addition, the UDVA recovery is also faster in the patients receiving RLE surgery [25], and a postoperative UDVA of 20/20 can be achieved in 65% of patients receiving RLE surgery [26]. Nevertheless, there was no study that evaluated the efficiencies of RLE and KLEx surgeries in the same population. To our knowledge, this study may be the first to exhibit a comparison of postoperative UDVA recovery between the RLE surgery and the KLEx surgery. In addition, the myopia and astigmatism degrees of the two groups were similar; thus, the influence of refractive error extents on the whole-group analysis of this study may be minor. In addition, we adjusted for age and sex in the multivariable analysis that evaluated the trends in visual recovery from the two surgeries. Consequently, the compatible results of visual recovery of the RLE and KLEx surgeries may be credible. The second-generation KLEx surgery may have resulted in a relatively limited UDVA improvement one day postoperatively due to corneal edema resulting from the high-frequency laser emission and more laser spots [19], but the UDVA reached an acceptable level one week postoperatively in our patients. The UDVA of the RLE group also presented a smooth recovery, in which the mean UDVA one day postoperatively was 0.24. The relatively worse postoperative UDVA in the RLE group than in the second-generation KLEx surgery group may have resulted from the lower level of initial postoperative UDVA in the RLE group. The UDVA recovery resulting from the RLE surgery was not inferior to that from the second-generation KLEx surgery. The difference in percentages of patients reaching a final UDVA of 20/20 between groups was about 9 percent, which showed minimal statistical and clinical differences.

The postoperative refractive status of the RLE and KLEx groups were similar concerning the amount of SE, while the RLE group experienced a slightly higher degree of SE fluctuation. The RLE surgery can be arranged for individuals with extreme myopia, and some individuals may receive the RLE surgery due to an inadequate CCT for corneal refractive surgeries [27]. In this study, the individuals who received RLE surgery presented with a myopia degree ranging from −4.00 D to −9.50 D, which is similar to the myopia degree of those who received second-generation KLEx surgery in the current study. The SE values of the RLE group and the KLEx group were similar from the day after the surgery until the final visit in the current study, which indicates that the refraction stabilities of the two refractive surgeries are comparable. Also, the ratios of individuals who achieved final SE results of ±−0.50 D and ±−1.00 D were similar between the RLE group and the KLEx group, which indicates that the predictabilities of the two refractive surgeries are similar. Furthermore, the vector analysis demonstrated similar SIA, TIA, and AE results between the RLE group and the KLEx group. The above astigmatic parameters indicate that the astigmatism correction results of the two refractive surgeries were similar. The DV and ME were significantly lower in the KLEx group, which is approximately 60 percent of the results in the RLE group. The intraocular lens implanted during RLE would rotate even after the surgery [28], and the second-generation KLEx surgery has an eye-tracking system that can target the corneal apex and, thus, may reduce the amount of residual astigmatism [20]. The above phenomenon may be the reason for the lower DV and ME in the KLEx population than in the RLE population. Still, the difference in the CoI was about 0.06 between groups, which is not a prominent amount in clinical practice; thus, the difference in astigmatism correction between the two surgeries may be minimal.

Regarding the postoperative outcomes in the individuals with different degrees of myopia, the UDVA one day postoperatively was numerically better in the high myopia individuals who received RLE surgery than in those who received second-generation KLEx surgery. On the other hand, the postoperative UDVA was numerically better in the low myopia population who received second-generation KLEx surgery than in those who received RLE surgery. In a previous study, the postoperative UDVA in patients who received RLE did not differ among different baseline myopia degrees [29]. Regarding the KLEx surgery, the visual recovery after first-generation KLEx surgery was significantly faster in patients with low myopia than in the high myopia population: 34.2% of patients reached a 20/20 UDVA one day postoperatively in the high myopia group, which is significantly worse than the 50% in the low myopia group [30]. In an article analyzing the second-generation KLEx surgery, the percentage of postoperative UDVA results that reached 20/12.5 was also numerically better in the low myopia group than in their high myopia counterpart [17]. Thus, it is reasonable to conclude that the postoperative UDVA was slightly better in those who received RLE surgery than in those who received second-generation KLEx surgery in the high myopia population. The final postoperative UDVA and SE performances were numerically better in the low myopia population than in the high myopia population in both groups, while the final postoperative SE and UDVA results showed similar values between the RLE and KLEx groups with different myopia degrees. There was a rare publication that reported this phenomenon. Although the KLEx group yielded a higher percentage of UDVA and SE results that reached a specific threshold, the difference did not achieve statistical significance. Our findings may imply the high efficiency and predictability of the two refractive surgeries in different myopia degrees.

Concerning the postoperative outcomes of the second-generation KLEx surgery and RLE surgery in this study, compared to earlier studies, our UDVA results 3 months after the second-generation KLEx surgery delineated an analogous numerical result to the second-generation KLEx surgery results reported in an earlier article [24]. For postoperative refraction, 97.14 percent of subjects who accepted second-generation KLEx surgery delineated a postoperative SE result within ±1.00 D, and 88.57 percent of subjects who received second-generation KLEx surgery revealed a postoperative SE result within ±0.50 D, which is also not inferior to the postoperative SE evaluation in people who accepted refractive surgeries, according to previous articles [31,32]. Furthermore, the final UDVA result in the RLE group was 0.1, which is comparable to the results of individuals who accepted RLE surgery in previous studies [22]. Moreover, 94.74 percent of individuals who accepted RLE surgery delineated a postoperative SE within ±1.00 D, and 78.95 percent of individuals who accepted RLE surgery delineated a postoperative SE within ±0.50 D. Compared to a previous study, the results in the RLE population of this study also represent acceptable outcomes [25]. There were no severe complications in either the RLE group or the KLEx group of this study. Approximately five patients in each group experienced irritation and dryness after the surgery, but none of them complained about these symptoms one month postoperatively. Accordingly, the surgical quality of both refractive surgeries performed in our institution may be acceptable. The rates of retinal detachment after clear lens extraction were above 5 percent in earlier publications [33,34], while the incidences have decreased in the recent literature, and several studies reported the absence of retinal detachment after clear lens extraction/RLE [22,25,35]. Combined with the results of this study (i.e., no newly developed retinal degeneration, retinal break, or retinal detachment episodes after both surgeries), the safety of RLE might be acceptable.

There were some limitations in this study. Firstly, the retrospective nature of this study could have reduced the homogeneity of the study population compared to a prospective randomized trial. Furthermore, the small number of subjects in this study, in which only 54 eyes were analyzed, could negatively impact the statistical integrity of this study's results, although the statistical power of our study population was not very low. Also, we did not analyze visual dysphotopsias such as glare, halos, starbursts, and other vision problems due to the retrospective design, and the study population was not randomized. Because of the small case numbers and retrospective nature, our findings may serve as a preliminary study for a subsequent prospective one. Moreover, the RLE and KLEx surgeries were performed by two different surgeons, and the choice of target refraction and surgical technique may be different between the two ophthalmologists. Finally, all the individuals included in this study were Han Taiwanese, and, thus, the external validity of this study was diminished.

## 5. Conclusions

In conclusion, the efficiency and predictability of the RLE surgery and second-generation KLEx surgery were comparable. Furthermore, the second-generation KLEx surgery may have a certain advantage regarding astigmatism correction. Consequently, the second-generation KLEx surgery may be recommended to people with high astigmatism and adequate corneal thickness. Further large-scale prospective studies to survey whether the predictors for better postoperative outcomes are different between the RLE and KLEx surgeries is mandatory.

## Figures and Tables

**Table 1:** Basic characters of the two groups.

Characters	RLE Group (*N* = 19)	KLEx Group (*N* = 35)	*p*
Age (range)	48.29 ± 8.37 (39–57)	42.53 ± 7.52 (36–50)	0.019 *
Sex (male:female)	12:7	22:13	0.610
Systemic disease			0.302
Hypertension	1	0	
Diabetes mellitus	0	1	
Others	0	0	
CDVA (LogMAR)	0.05 ± 0.06	0.01 ± 0.03	0.016 *
Cycloplegic refraction (D)			
Sphere	−6.85 ± 2.97	−6.43 ± 2.57	0.618
Cylinder	−1.54 ± 0.35	−1.62 ± 0.27	0.384
SE	−7.62 ± 2.83	−7.24 ± 2.40	0.655
Topographic cylinder (D)	−1.82 ± 0.46	−1.96 ± 0.39	0.287
Sim K	43.29 ± 2.15	42.88 ± 2.24	0.578
CCT (μm)	548.32 ± 25.76	542.51 ± 24.48	0.472
Angle kappa	0.14 ± 0.11	0.15 ± 0.10	0.763
Pupil diameter (mm)	4.93 ± 1.14	4.77 ± 1.28	0.685
Schirmer test (mm)	11.24 ± 3.34	12.03 ± 2.69	0.384

CDVA: corrected distance visual acuity, CCT: central corneal thickness, K: keratometry, KLEx: keratorefractive lenticule extraction, *N*: number, RLE: refractive lens exchange, SE: spherical equivalent. * denotes significant difference between the two groups.

**Table 2:** The postoperative outcomes at the final visit of the two groups.

Outcome (%)	RLE Group	KLEx Group	*p*
UDVA (LogMAR)			
≥20/25	84.21% (16)	85.71% (30)	0.882
≥20/20	73.68% (14)	82.86% (29)	0.323
SE			
≥±0.50 D	78.95% (15)	88.57% (31)	0.285
≥±1.00 D	94.74% (18)	97.14% (34)	0.584

KLEx: keratorefractive lenticule extraction, RLE: refractive lens exchange, SE: spherical equivalent, UDVA: uncorrected distance visual acuity.

**Table 3:** The vector analysis of astigmatism of the two groups.

Parameter	RLE Group	KLEx Group	*p*
TIA	−1.48 ± 0.31	−1.53 ± 0.25	0.566
SIA	−1.28 ± 0.27	−1.40 ± 0.20	0.079
DV	0.45 ± 0.15	0.29 ± 0.11	<0.001 *
ME	−0.20 ± 0.12	−0.13 ± 0.09	0.029 *
AE	3.97 ± 19.21	2.26 ± 11.89	0.716
CoI	0.86 ± 0.05	0.92 ± 0.03	<0.001 *

AE: angle of error, CoI: correction index, DV: difference vector, KLEx: keratorefractive lenticule extraction, ME: magnitude of error, RLE: refractive lens exchange, SIA: surgically induced astigmatism, TIA: target-induced astigmatism. * denotes a significant difference between groups.

**Table 4:** The postoperative outcomes at the final visit in different subgroups.

Subgroup	RLE Group	KLEx Group	*p*
High myopia (10, 18)			
UDVA ≥ 20/25	80.00% (8)	83.33% (15)	0.601
UDVA ≥ 20/20	70.00% (7)	77.78% (14)	0.491
SE ≥ ±0.50 D	70.00% (7)	83.33% (15)	0.358
SE ≥ ±1.00 D	90.00% (9)	94.44% (17)	0.595
Low myopia (9, 17)			
UDVA ≥ 20/25	88.89% (8)	88.24% (15)	0.732
UDVA ≥ 20/20	77.78% (7)	88.24% (15)	0.431
SE ≥ ±0.50 D	88.89% (8)	94.12% (16)	0.582
SE ≥ ±1.00 D	100.00% (9)	100.00% (17)	0.999

KLEx: keratorefractive lenticule extraction, RLE: refractive lens exchange, SE: spherical equivalent, UDVA: uncorrected distance visual acuity.

## Data Availability

The data used in this study are available from the corresponding author upon reasonable request.
